# Non-Invasive Screening Tools for Down’s Syndrome: A Review

**DOI:** 10.3390/diagnostics3020291

**Published:** 2013-05-31

**Authors:** Kelly A. Sillence, Tracey E. Madgett, Llinos A. Roberts, Timothy G. Overton, Neil D. Avent

**Affiliations:** 1 School of Biomedical and Biological Sciences, Plymouth University Peninsula School of Medicine and Dentistry, Plymouth University, Plymouth, PL4 8AA, UK; E-Mails: kelly.sillence@plymouth.ac.uk (K.A.S.); tracey.madgett@plymouth.ac.uk (T.E.M.); llinos.roberts@plymouth.ac.uk (L.A.R.); 2 Department of Obstetrics, St Michael’s Hospital, Southwell Street, Bristol, BS2 8EG, UK; E-Mail: timoverton@me.com

**Keywords:** screening, Down’s syndrome, non-invasive, biomarkers, sonographic markers, next-generation-sequencing

## Abstract

Down’s syndrome (DS) is the most common genetic cause of developmental delay with an incidence of 1 in 800 live births, and is the predominant reason why women choose to undergo invasive prenatal diagnosis. However, as invasive tests are associated with around a 1% risk of miscarriage new non-invasive tests have been long sought after. Recently, the most promising approach for non-invasive prenatal diagnosis (NIPD) has been provided by the introduction of next generation sequencing (NGS) technologies. The clinical application of NIPD for DS detection is not yet applicable, as large scale validation studies in low-risk pregnancies need to be completed. Currently, prenatal screening is still the first line test for the detection of fetal aneuploidy. Screening cannot diagnose DS, but developing a more advanced screening program can help to improve detection rates, and therefore reduce the number of women offered invasive tests. This article describes how the prenatal screening program has developed since the introduction of maternal age as the original “screening” test, and subsequently discusses recent advances in detecting new screening markers with reference to both proteomic and bioinformatic techniques.

## 1. Introduction

**Figure 1:**
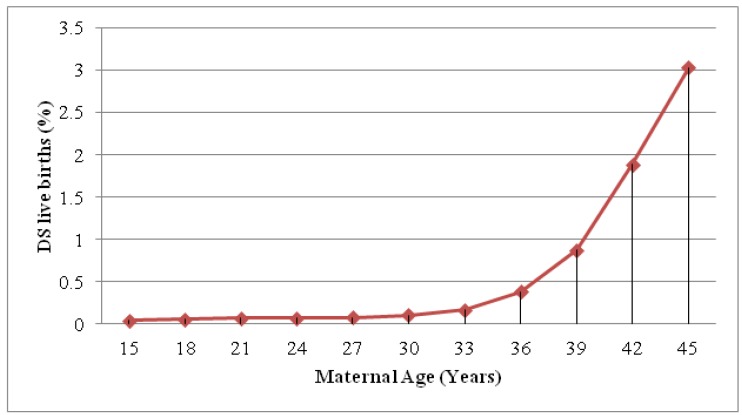
The estimated risk of DS according to maternal age (adapted from [3]).

Down syndrome (DS) is the most common chromosomal aneuploidy and is the leading genetic cause of developmental delay. The overall incidence of DS is around 1 in 800 live births [1,2], but the risk of fetal trisomy is directly related to maternal age, increasing gradually up to age 33 and subsequently increasing exponentially (Figure 1). Women in their late 40s have an incidence rate of around 1 in 32 live births [3]. Between 1989 and 2008, the percentage of women conceiving aged 35 years and over increased from 9% to 20%, respectively, which led to a 71% rise in the number of DS pregnancies [2,4]. Despite an expected 1.32 fold increase in the number of DS live births as a result of this, the reported rate in England and Wales fell by 1% from 736 live births in 1989 to 750 live births in 2008 [2,5]. In the UK the National Down’s Syndrome Cytogenetic Register (NDSCR) indicated that without improved screening tools between 1989 and 2008, the continuous rise in maternal age would have caused a 48% increase in live births with DS [3]. Although there are clear ethical issues surrounding prenatal screening, with the majority of women terminating affected pregnancies, the evidence provided clearly illustrates the effectiveness of screening for DS.

In addition to advanced maternal age, other risk factors include previous family history and gestational age, as 43% of DS pregnancies miscarry between 10 weeks and term [6,7]. The gradual introduction of various biochemical and sonographic markers since the early 1980s, has greatly improved the sensitivity of current screening programs to around 95% [6]. Women with a high risk following screening are offered invasive procedures such as amniocentesis or chorionic villus sampling (CVS) for a definitive diagnosis. However, these invasive procedures are associated with around a 1% risk of iatrogenic fetal loss [1,8,9]. Advances in screening tools could further improve the specificity and sensitivity of current screening methods, thus reducing the number of women offered invasive diagnostic tests. In spite of the huge recent advances in non-invasive prenatal diagnostics using next generation sequencing (NGS) [1], screening will remain an essential first line test in the clinical management of aneuploid pregnancies. This review will outline the development of screening over the last four decades up to present day and discuss possible new screening tools that could potentially be used in a clinical setting. 

## 2. Definitions

There are various measurements that can be used to determine the success of a screening program including; the detection rate (DR), the false positive rate (FPR), screen positive rate (SPR), and the odds of a positive result (OAPR). The DR (sensitivity) of the test identifies the proportion of affected cases successfully identified by the screening program, for example a DR of 90% means that the screening test will successfully detect 9 out of 10 cases of DS. However, high sensitivity alone is not sufficient for DS detection. The test must also display a low FPR, which is defined as the rate of occurrence of positive results in non-affected cases. More recently, the SPR has been used as an alternative to the FPR. The screen positive rate identifies those with a result above the cut off risk (for example 1 in 150) and will include both true positives and false positives [5]. It is important that the FPR/SPR is kept as low as possible so to minimize the number of women offered invasive procedures which will in turn reduce the number of miscarriages of healthy fetuses. The likelihood of a woman having a DS pregnancy confirmed by CVS or amniocentesis if her screen risk is high is known as the OAPR. If a screening test has a high OAPR, more affected pregnancies will be successfully diagnosed for every miscarriage caused by invasive testing [10,11]. Both the DR and the FPR/SPR are influenced by the risk threshold above which invasive testing is offered. In an ideal screening test the DR would be high (>90%) and the SPR would be low (<2%). However, increasing the threshold (for example to 1 in 100) would cause both the DR and the SPR to decline, and decreasing the threshold (for example to 1 in 300) would cause both the DR and SPR to increase [5]. 

**Figure 2:**
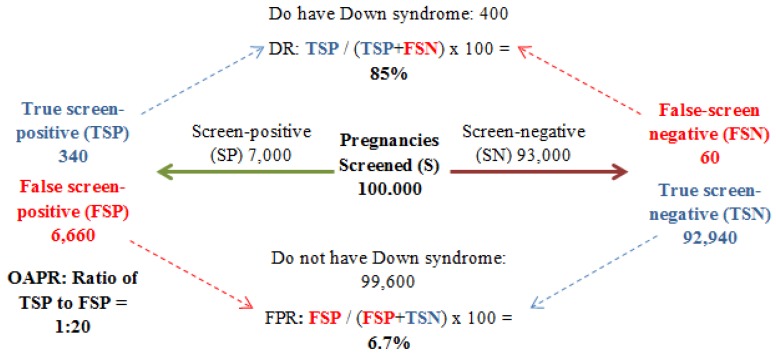
The screening process, potential outcomes and measures of accuracy. Detection rate (DR): Proportion of affected cases successfully identified by the screening test. TSP/(TSP + FSN) = 85%. False positive rate (FPR): Proportion of positive results in non-affected cases identified by the screening test. FSP/(FSP + TSN) = 6.7% (adapted from [10,11]).

Since the introduction of screening for DS the DR has greatly improved parallel to a decrease in the SPR [10]. Therefore more affected cases of DS are being detected via screening and fewer non-affected cases are being identified as high risk. This has led to an overall increase in OAPR and consequently a decline in the number of invasive tests offered to women. Although there is still room for improvement, Figure 2 illustrates the possible outcomes and measures of accuracy of the screening process [11].

## 3. Screening: Past to Present

### 3.1. Historical Overview

In the early 1980s, maternal age was effectively the only screening tool available for detection of DS and invasive diagnostic tests were offered to all women aged 35 years and above. These tests were only offered to women younger than 35 years if there was known family history of the disorder [12]. However this approach was inappropriate and unsustainable for numerous reasons. Firstly, maternal age alone is not an effective screening test as it has a DR of less than 35%, meaning that most fetuses with DS were undetected and many women with unaffected fetuses were subjected to unnecessary invasive testing [5,13]. Secondly, as the average maternal age was beginning to rise, resources to perform invasive testing for all these women were unavailable [5].

To improve the sensitivity of screening for DS, sonographic and biochemical screening tests were developed that could be combined with maternal age to increase the accuracy of risk assessment. The initial opportunity to improve screening arose in 1984, when several studies identified an association between low alpha-fetoprotein (AFP) levels (around a 25% reduction) in maternal serum and fetal aneuploidy [14,15,16]. AFP is a large serum glycoprotein produced by both the yolk sac and the fetal liver, and is considered to function in a similar way as albumin in adults [17]. DiMaio *et al.* identified that using a cut-off for risk at which 5% of women under 35 are offered invasive testing, around 25–30% of pregnancies in which the fetus has DS will be detected using AFP serum biomarker alone [18]. The identification of this marker for DS detection was a serendipitous scientific discovery, initially raised AFP levels were used to identify pregnancies that were potentially affected by fetal neural tube defects, particularly anencephaly, it was only during this cohort that the link between low AFP levels and an increased incidence of DS was identified. Now AFP is used clinically worldwide for screening of DS after the first trimester as one of the biochemical serum markers used in the quadruple test. 

Since then, various pregnancy-associated maternal serum markers for DS have been evaluated. Key markers that have been incorporated into the screening program include human chorionic gonadotropin (hCG), estriol, inhibin A and pregnancy associated plasma protein A (PAPP-A). hCG is a hormone initially produced by the embryo and later by the syncytiotrophoblast. Its function is to enable the secretion of progesterone, which promotes the maintenance of the corpus luteum [19]. During very early pregnancy hCG levels increase rapidly until 12 weeks gestation, at which point the hCG levels off, normal hCG values during the second trimester range between 4,060 and 165,400 IU/L. In 1987, Bogart *et al.* identified an association between an increase in serum levels of hCG and DS pregnancies (approximately double the normal values), which led to the introduction of the second trimester double test a year later in the UK [20]. This test measured maternal serum concentrations of both AFP and hCG between 15 and 20 weeks gestation alongside maternal age. With a risk threshold of 1 in 250, the DR was approximately 60% with a SPR of 5% [5]. 

Shortly after the double test was established in the UK, studies reported a 25% reduction of unconjugated estriol in DS pregnancies (normal value at 15 weeks gestation is around 4 nmol/L) [21]. The addition of estriol as a third marker was the basis for the “Triple test’’ [22,23]. In the early 1990s the triple test was adjusted by the replacement of hCG with the free beta subunit of hCG (fβ-hCG) as it is this which is more markedly increased in DS pregnancies [24]. Although the triple test was associated with higher sensitivity (67% DR), it was not considered to be a great improvement on the double test, as the SPR was not lowered and the costs of screening were increased [25]. However, in the early 1990s, inhibin A was found to be significantly elevated in DS pregnancies, leading to the generation of the quadruple test with an improved DR of 75% [26]. The double, triple and quadruple test all offer a greater DR than maternal age alone but can only be performed during the second trimester. 

In 1991 maternal serum associated plasma protein-A (PAPP-A) was shown to be reduced by around 50% in DS pregnancies and was detectable from as early as 8 weeks gestation [22,27]. Between 17 and 19 weeks gestation maternal serum PAPP-A levels in DS affected pregnancies returned to those values observed with unaffected pregnancies [28,29]. Throughout the 1990s the emphasis was to perform screening in the first trimester, allowing parents to decide at an earlier stage in the pregnancy whether to undergo invasive testing. 

In addition to these biochemical markers, the risk of DS pregnancies can also be evaluated by the identification of physical markers using sonographic imaging. In 1992 the ultrasound screening test of nuchal translucency (NT) was developed by Nicolaides *et al.* [30], the ultrasound NT is the sonographic appearance of a collection of fluid under the skin behind the fetal neck in the first trimester between 11 and 13 weeks gestation. The maturation of the fetal lymphatics often occurs later during the second trimester in fetuses with DS and other chromosomal abnormalities, which causes an increase in fluid collection.

During the early 1990s a number of reports identified an association between DS and increased NT.In 1994, Nicolaides *et al.* reported that an NT value ≥2.5 mm was seen in 84% of fetuses with DS and 4.5% of euploid fetuses in a study involving 1,273 pregnancies [31]. However, it is important that when measuring the NT thickness care is taken when aligning the calipers, as an error of 0.4 mm can significantly alter the risk. For example, at 12 weeks gestation the risk of having a DS fetus when NT values of 2.6 mm and 3.0 mm are recorded is quoted as 1 in 1,394 and 1 in 563, respectively [32]. When maternal age alone was used as a screening tool, only two out of 11 cases of DS were detected, however following the introduction of NT measurement, three out of four cases of DS were detected by karyotyping because of an increased NT, this illustrates that when obtained by well-trained professionals, NT measurement is a highly reproducible screening tool [33,34]. 

**Figure 3:**
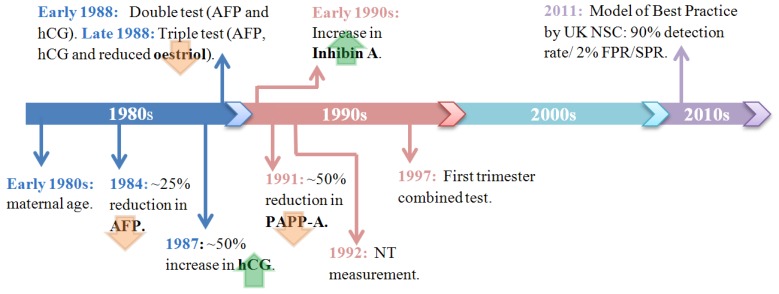
Timeline summarising the key developments in UK DS screening, from the early 1980s when maternal age was effectively the only screening tool used up to the identification of the Model of Best Practice (MoBP) identified by the UK National Screening Committee (NSC) in 2011.

The combination of NT, maternal age and early detectable serum biomarkers (fβ-hCG and PAPP-A) was referred to as the first trimester combined test [35]. Studies have identified that with the first trimester combined test around 85–90% of all DS cases could be detected with a 5% FPR [36,37,38,39]. Figure 3 illustrates a short summary of key DS screening developments incorporated in a clinical setting from the early 1980s to date. 

### 3.2. Current Methods

In 2008 the UK National Screening Committee’s (UK NSC) Model of Best Practice (MoBP) for DS screening set a target for 2010/11 to achieve a DR of 90% and a FPR/SPR of 2%, however this is yet to be achieved (Figure 3). The test currently closest to achieving standards set by the MoBP is the first trimester combined screening test (DR 85–90% and FPR 5%). However, women who miss first trimester screening can only be offered second trimester quadruple testing, which has a slightly lower sensitivity (75% DR) and a higher FPR (6.9%) than the first-trimester combined test [5].

Some hospitals also offer the integrated test [40], which is performed in two stages. Firstly the combined test is performed followed by second-trimester biochemistry (quad test) a few weeks later [41]. This test is used to help reduce the FPR, as women that are high risk following the combined test may become a low risk following the result of the integrated test. In 2013 the International Society for Prenatal Diagnosis (ISPD) identified that integrated screening can be offered when CVS is not available [41]. However, the UK NSC does not recommend integrated testing for two fundamental reasons. Firstly, a woman who is considered to be high risk following the combined test may not return for her quad test and therefore may be lost within the system without having been counseled properly and secondly, there are higher cost and service implications associated with combining the two screening tests [42]. A possible compromise to this problem is Contingency screening, which allows pregnant women with a significantly high risk following first-trimester screening to be offered invasive diagnostic tests immediately. In contrast, pregnant women that indicate extremely low risk after first trimester screening are reassured. It is only those women with an intermediate risk value (between 1 in 50 and 1 in 1,000) that are offered further testing with other ultrasound markers including nasal bones, tricuspid regurgitation and ductus venous Doppler to further refine the risk before offering invasive testing. This approach results in a DR of 90% for a FPR of 3% [37]. Currently, the UK NSC has also not supported the Contingency screening test despite improvements to DR and FPR/SPR, because of the complexity associated with the technique and the implications for service reconfiguration [5].

In the United States, the Society for Maternal-Fetal Medicine (SMFM) completed a survey in 2007 to determine changes in screening and numbers of invasive diagnostic procedures performed since 2001. The results showed that over this time frame the evolution and increased uptake of DS screening between 2001 and 2007 led to a 20% reduction in invasive diagnostic procedures [43]. The ISPD recognizes that the use of maternal age alone to assess fetal DS risk in pregnant women is insufficient and has stated that a combination of ultrasound NT measurement and serum markers in the first trimester should be available to all women who desire early risk assessment. For women that first attend their prenatal care after 13 weeks 6 days gestation, the ISPD recommends that the quadruple test should be provided [44].

## 4. Further Developments

Since the early 1980s enormous progress for DS screening has been made, however further improvements are still required. The problem associated with current screening tests is that 5% or more of screened women need to undergo invasive testing in order to detect 60–80% of fetuses with DS, resulting in large numbers of false screen-positives. In 2008 it was estimated that approximately 400 babies without DS were miscarried following invasive procedures on women with false positive screening results in England and Wales [11]. Here we look at new screening techniques that are being developed that could potentially raise sensitivity of current screening methods (to >90% DR) and lower the FPR/SPR (to <2%), allowing more DS cases to be detected and less invasive testing to be offered, thus reducing the number of miscarriages in affected and unaffected pregnancies. 

### 4.1. Sonographic Markers of DS

The role of sonographic markers in the risk assessment of DS has been extensively investigated at the 11–14 week scan and at the time of the mid-trimester fetal anomaly scan. Sonographic markers at the 11–14 week scan include structural abnormalities (exomphalos, cystic hygroma, *etc*.) and more subtle markers such as presence or absence of nasal bones, tricuspid regurgitation and reversed flow in the ductus venosus. Markers at the mid-trimester scan can again be divided into structural anomalies (congenital heart disease, anterior abdominal wall defects, ventriculomegaly, *etc*.) and more subtle markers (choroid plexus cysts, echogenic foci in the heart, increased nuchal fold, *etc.*) traditionally referred to as “soft markers”. 

The association between structural anomalies and aneuploidy detected during the first trimester or mid-trimester scan is well established. Fetal exomphalos or Fallot’s tetralogy for example has a significant association with DS. Detection of structural anomalies at the time of either the 11–14 week scan or the mid-trimester scan should lead to the offer of amniocentesis or CVS. Atrioventricular septal defects (AVSD) are an example of second-trimester structural anomaly. In pregnancies that demonstrate a normal fetal karyotype, the frequency of AVSD is 1 in 10,000 live births, but in DS pregnancies this increases significantly to 2,000 in 10,000 live births (1 in 5 incidence) [45]. However, repeated studies have shown that less than 25% of affected fetuses demonstrate major structural abnormalities, whereas 1 or more “soft markers” could be observed in 50% or more cases [46,47,48].

**Figure 4:**
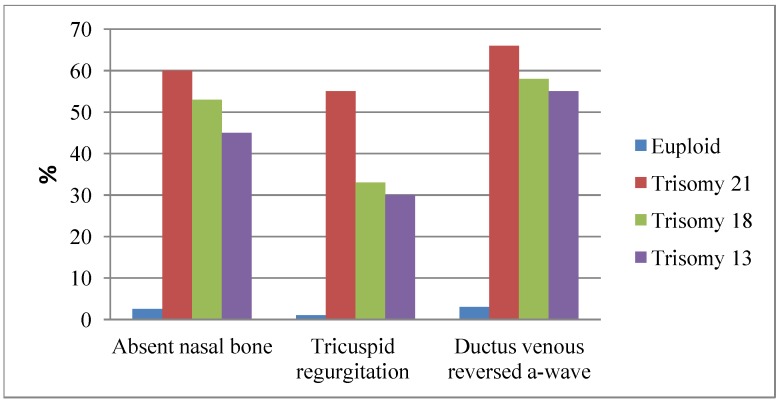
Sonographic features of trisomies 21, 18 and 13 (adapted from [50]).

The presence or absence of the more subtle features at the 11–14 week scan has been used to refine the risks generated by combined screening. Hypoplasia of the nasal bone is identified in 65% of fetuses with DS between 11 and 14 weeks gestation. However, this marker shows significant inter-racial variation. In Caucasian populations only 1–3% of euploid pregnancies have an absent nasal bone during late first-trimester whereas in African populations this increases to around 10% [5].Incorporating nasal bone assessment into combined screening therefore gives better results in Caucasian populations. Doppler flow examination across the tricuspid valve and in the ductus venosus, have also proved useful markers. In 2009, Kagan *et al.* performed a large scale study involving 20,000 euploid pregnancies which included 122 cases with DS. Reversed flow in the a-wave of the ductus venosus and tricuspid regurgitation were observed in 55% and 60% of DS cases, and in 3.2% and 0.9% of euploid cases, respectively [49]. Incorporation of these markers into a first-trimester combined screening test can increase the DR to 93–96% with a FPR of 2.5% [47]. Figure 4 illustrates the occurrence of these sonographic features in euploid and trisomy foetuses. Checking for these additional markers is not only challenging but also very time- consuming and they have not been adopted into routine clinical practice for widespread screening. They may have a role, however, in a contingent screening model, whereby they are offered to women with an intermediate risk from combined screening who need further information before deciding whether to opt for invasive testing [37].

The significance of the identification of soft markers at the time of the mid-trimester scan has been far more contentious. In the 1990s it was common place for women to be offered invasive procedures when choroid plexus cysts, echogenic foci in the fetal heart, mild renal pelviceal dilatation were noted at the time of the 20 week scan. However, a review of the importance of these soft markers in 2001 confirmed their very low sensitivity and specificity for DS with the exception of an increased nuchal fold (the thickness of skin at the back of the fetal neck noted at the time of the mid-trimester scan, not to be confused with nuchal translucency measurements at the time of the first trimester scan) which had a likelihood ratio of 17 for DS [51]. One of the reasons why the importance of soft markers has diminished is because of the widespread adoption of first and second trimester screening over the last 10 years. Poor uptake in screening in the early 1990s meant that the prevalence of DS at the time of the mid-trimester scan was much greater than in current practice. Screening tests perform better when the prevalence the condition being screened for is high. With the increasing uptake of effective DS screening before 20 weeks the efficacy of screening using soft markers is now much less. 

A combination of these factors led to the National Screening Committee in the UK in 2009 recommending that the a priori risk for DS should not be adjusted depending on the presence or absence of single or multiple soft markers (choroid plexus cysts, dilated cisterna magna, echogenic cardiac foci and a 2 vessel cord).

### 4.2. New Serum Biomarkers

Despite recent advances in ultrasound technology allowing current screening techniques to achieve detection rates >90% with FPRs <5%, improvements to these rates is still a priority for current research in prenatal assessment. In addition to identifying new possible ultrasound markers, novel biochemical screening markers to improve current DRs and FPRs/SPRs have been extensively studied [52,53,54,55,56,57,58]. Since the discovery of cell-free fetal DNA (cffDNA) within the maternal circulation many advances have been made in prenatal screening [52]. Recent studies exploring the proteomic profile of maternal serum have identified both non-epigenetic and epigenetic screening markers that could potentially be used as an alternative or in addition to current screening tools to provide greater specificity and lower FPRs/SPRs [53,54,55,56,57,58]. The SAFE (Special Non-Invasive Advances in Fetal and Neonatal Evaluation) NoE (Network of Excellence) was established by the European Union (EU) in 2004 to implement routine, cost-effective NIPD and neonatal screening through the formation of long term partnerships worldwide [59,60]. The program played a key role in the standardization of RhD genotyping, and also set out to identify a panel of new, more informative, biomarkers for fetal DS detection. Despite the program ending in March 2009, the long term goals set out by the SAFE NoE are still a key area of research [59].

Non-epigenetic markers, such as maternal serum markers (MSMs) used in the combined screening test, simply show a marked increased or decreased level in affected cases in comparison to euploid pregnancies. Novel biochemical markers are currently under investigation but so far there has been no formal large scale evaluation of new markers by the UK NSC to inform policy. Epigenetic approaches have also been examined in an attempt to discriminate the fetal DNA molecules from the high background of maternal DNA fragments (around 90% of total DNA). Difference in DNA methylation between the mother and fetus is currently the most characterized epigenetic modification studied for possible prenatal detection of DS [61,62]. Targeting fetal-specific markers allows for the generated signal to be completely fetal in origin, subsequent chromosomal dosage can then be carried out for trisomy identification. Table 1 illustrates various studies over the past few years that have published results on potential new biomarkers (both non-epigenetic and epigenetic) that could be used to improve the sensitivity of current screening programs. For both PlGF and ADAM12 (Table 1) detection needs to occur prior to 10 weeks gestation, as they are both almost non-existent by this time. Though it would be ideal to screen for DS this early in pregnancy, these tests are fairly unpractical because women have often not had their first pregnancy appointment with either their doctor or midwife. However if early screening is possible it has been identified that the addition of PlGF to the combined test can help to increase the DR by 4–7% [63]. Alternatively, the results for the CA15-3 and CA19-9 (Table 1) were not affected by maternal age [54]. Kamyab *et al.* identified that both the accuracy and specificity were improved by using two target genes (*DSCAM* and *DYRK1A*), producing an overall specificity of 96% and sensitivity of 80% [56]. Providing further validation studies are carried out it is possible that these biochemical markers may help to improve current screening tests.

**Table 1:** Summary of studies identifying potential new biochemical markers for prenatal screening of DS.

*Non-Epigenetic Markers*			
Study	Marker	Assay	Results
Cowens *et al.* [54]	Placental growth factor (PlGF)	DELFIA Xpress immunoassay platform.	Increase during early first trimester in affected DS pregnancies (1 MoM in unaffected pregnancies, 1.3 MoM in DS pregnancies, *p* < 0.0001).
Wang *et al.* [64]	ADAM12	Auto DELFIA/DELFIA ADAM12 Research kit (PerkinElmer Life and Analytical Sciences, Finland).	Reduction during early first-trimester in affected DS pregnancies (1 MoM in unaffected pregnancies, 1.26 MoM in DS pregnancies, *p* < 0.05).
Akinlade *et al.* [55]	CA15-3C A19-9	Quantified by the Kryptor Analyzer.	No difference between euploid and DS pregnancies. Significantly elevated in DS pregnancies. (0.98 MoM in euploid, 1.16 MoM in trisomy 21, *p* = 0.024).
Kamyab *et al.* [56]	*DSCAM DYRK1A*	Multiplex assay with cytogenetic analysis and QF-PCR.	The mean gene dosage rate was significantly increased for both genes in DS pregnancies compared to euploid pregnancies (*p* < 0.001).
***Epigenetic Markers***			
Lim *et al.* [57]	*PDE9A*	Quantitative methylation specific-PCR.	*M-PDE9A* (maternal) did not differ between pregnancies, but levels of *U-PDE9A* (fetal) were significantly higher in DS pregnancies.
Du *et al.* [58]	*DSCR4*	Methylation specific primers and digital PCR.	Hypomethylated in placental tissue and methylated in maternal cells. Can detect and quantify unmethylated *DSCR4* in the first-trimester maternal plasma, successfully detect DS by RCD against a reference gene (e.g., *ZFY*).
Chim *et al.* [65]	*SERPINB5* (coding for Maspin)	Bisulphite genomic sequencing and RT-Quantitative methylation-specific PCR.	Hypomethylated in placental tissue and methylated in maternal cells. *SERPINB5* was the first fetal-specific hypomethylated gene to be identified in maternal plasma.

The phosphodiesterase gene, *PDE9A,* is an example of an epigenetic marker, as it is completely methylated in maternal blood (*M-PDE9A*) and unmethylated in the placenta (*U-PDE9A*). In 2011, Lim *et al.* report a DR of 77.8% of DS pregnancies for this marker and a 5% FPR, demonstrating that *U-PDE9A* is an effective biomarker for the non-invasive diagnosis of DS during the first-trimester of pregnancy [57]. Other studies have also identified epigenetic markers (Table 1) for DS screening, but before any can be approved by the UK NSC, large validation studies must be carried out. 

Currently there are many developments occurring in integrated proteomics and bioinformatics analysis in an attempt to identify multiple candidate protein biomarkers from maternal serum for detection of DS. Kang *et al.* identified 31 DS differentially expressed maternal serum proteins (DS-DEMSPs) using the latest proteomic techniques to identify proteins differentially expressed in the maternal serum of women carrying a DS fetus, ten of which were considered as potential biomarkers (Alpha-2-macroglobulin, Apolipoprotein A1, Apolipoprotein E, Complement C1s subcomponent, Complement component 5, Complement component 8, alpha polypeptide, Complement component 8, beta polypeptide and Fibronectin) [66]. Initial bioinformatics analysis funded by SAFE NoE has identified differences of known placental and DS markers, such as genes located in the *DSCR* region of chromosome 21. The SAFE project identified that the combination of both bioinformatics and proteomic approaches could be used to find previously unidentified biomarkers of aneuploidy [59]. The integration of proteomics and bioinformatics would not only provide a useful tool for prenatal screening of DS, but would also provide a mechanism for the detection of other birth defects or pregnancy related disorders. However, is important to appreciate that plasma proteomics is extremely complicated due to the huge “noise” present when looking for new screening targets. Only a small number of studies have attempted to identify new biomarkers for DS, therefore it is essential that larger scale studies are conducted using newer technology, such as liquid chromatography-mass spectrometers, which can identify larger numbers of peptides in one analysis with great sensitivity [67]. It is likely however, that with the rapid advances in DNA technology developments in this area will be somewhat marginalized. 

### 4.3. Digital PCR and Next Generation Sequencing (NGS)

Since the identification of cffDNA in maternal plasma [52], the goal is to detect DS and other aneuploidy disorders, such as trisomy 18 (Edwards syndrome), Trisomy 13 (Patau syndrome) and Monosomy X (Turner syndrome), using NIPD. Unlike screening, NIPD does not identify the risk of DS but allows for a definitive diagnosis. Currently, cffDNA has allowed for successful NIPD of gender determination [68] and RhD status [69,70], and is available on a research basis for some single gene disorders such as sickle cell anemia [71]. Recently, studies have identified new sophisticated analytical methods, such as digital PCR and massively parallel sequencing (MPS) (also known as NGS) which are capable of detecting chromosomal aneuploidy from maternal plasma [1,8,72,73,74]. However, until these techniques pass the scientific and regulatory hurdles required to be considered diagnostic they could potentially be used to significantly improve current screening strategies. 

There have been various molecular techniques developed for the non-invasive prenatal testing (NIPT) of fetal aneuploidy, which are allele dependent and labor intensive [75,76]. As maternal plasma only contains up to 10% cffDNA, to detect the presence of a DS fetus the screening test would need to be able to detect a 5% difference in plasma DNA concentrations for a sequence located on chromosome 21. In conventional real-time PCR, a difference of one cycle threshold (Ct) value corresponds to a 2-fold change in copy number, making it very difficult to detect a 1.5-fold increase in only 10% of the total DNA [77]. Digital PCR quantifies nucleic acids by counting amplification from single molecules [78], allowing copy number changes less than 2-fold to be easily detected. Digital PCR can be performed manually, but can be labor intensive and replication levels are limited by format of plate used (96 well or 384 well). Alternatives to the manual approach are now emerging. One of these methods is the use of microfluidic chips, which splits the original sample into 765 reaction chambers [79]. 

**Figure 5:**
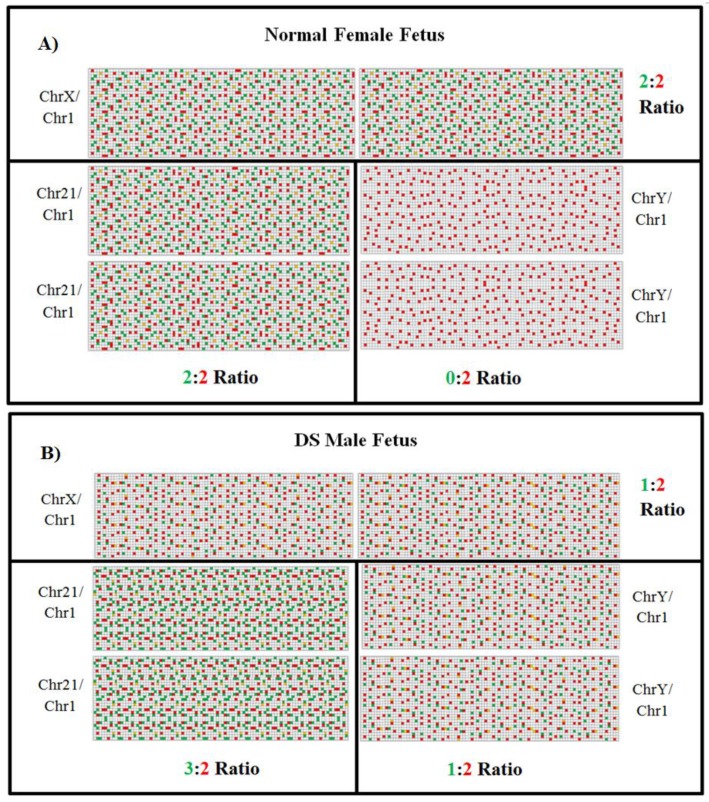
False-color images of microfluidic digital PCR chips. FAM signal is shown in green, which represents the target chromosome (chromosome X, Y or 21), and HEX signal is shown in red, which represents the reference chromosome (chromosome 1). Yellow squares indicate overlapping of HEX and FAM. (**A**) Euploid female fetus (46 XX). The ratio of chromosomes X and 21 are equal to reference chromosome 1 (2:2). There is no target Y chromosome identified. (**B**) DS male fetus (47 XY + 21). Ratio of chromosomes Y and X is half of reference chromosome 1 (1:2 ratios for X or Y and chromosome 1, respectively). This fetus indicates an increase of chromosome 21 in comparison to reference chromosome 1 (3:2 ratio, respectively), indicating trisomy 21 (adapted from [80]).

Figure 5, adapted from Fan *et al.* illustrates a mock microfluidic digital PCR chip image of a euploid female fetus and a DS male fetus. Detection of DS pregnancies can be identified by determining the allelic ratio. The ratio between the 21-target chromosome (FAM-labeled) against the reference chromosome 1 (HEX-labeled) is 3:2 and 2:2 in DS male fetus and euploid female fetus, respectively [80]. However, these results were achieved using CVS samples. To achieve this level of accuracy using maternal plasma samples is more challenging due to the high level of background maternal DNA. 

Microfluidic digital PCR does not rely on data that is collected during the exponential PCR phase and it does not require a standard for absolute quantification (unlike RT-PCR), which allows for improved precision and accuracy [81]. Lun *et al.* successfully detected fetal-derived Y-chromosomal DNA in maternal plasma using microfluidic digital PCR, which showed higher sensitivity compared with non-digital real-time PCR, 100% and 90%, respectively, and lower imprecision [82]. In 2007, Lo *et al.* identified an approach using digital PCR for the non-invasive detection of DS. Firstly, the report identifies a digital RNA-SNP strategy, which uses digital PCR to determine the imbalance of a single nucleotide polymorphism (SNP) on *PLAC4* mRNA, a placentally-expressed transcript on chromosome 21, in women bearing DS fetuses. Secondly, it identifies an alternative method known as the digital relative chromosome dosage (RCD) method. The RCD method is advantageous to the RNA-SNP approach as it does not require polymorphisms for analysis; it simply detects over- or underrepresented alleles by comparing copy numbers variation between chromosomes. However, DS could only be detected in samples containing 25% fetal DNA [83]. If a 25% fetal enhancement is achieved, 7,680 molecules would need to be analyzed to achieve successful characterization of trisomy status [84]. Evans *et al.* reported that if fetal DNA is enriched to 20%, then 2,609 counts would be sufficient to achieve a 99% DR for a 1% FPR. However, if fetal DNA is only enriched by 2%, over 110,000 counts would be needed to achieve a 95% DR for a 5% FPR [85]. Due to the high level of sensitivity achieved (99% DR), provided efficient prior-fetal enrichment, it is possible that digital PCR could potentially replace current screening methods. However, even though digital PCR could provide a cheaper alternative to NGS-NIPT, confirmation of the high-throughput possibilities and costs of digital PCR by large validation studies are still required.

The development of non-invasive tests based on cffDNA within the maternal circulation provides substantial new opportunities to improve prenatal screening. To date, the most convincing data for a generally applicable test for aneuploidy detection from cffDNA have been generated through MPS. This technology allows cffDNA obtained from maternal plasma to produce millions of short-sequence tags that can be aligned and uniquely mapped to a reference human genome that are by definition mapped to a specific chromosome [86]. The DR for fetal aneuploidy using this method is determined by the depth of sequencing and subsequent counting statistics. Fan *et al.* were the first to propose counting chromosomes using high-throughput massively parallel shotgun sequencing (MPSS) technology. In this study 5 million sequence tags were obtained per patient, providing sufficient data to detect the over- or under- representation of chromosomes and allow for correct classification of an aneuploidy fetus [87]. Table 2 illustrates the DR and FPR associated with large scale clinical trials of NIPT by MPS for fetal DS detection.

Ehrich *et al.* revealed that MPSS managed to detect all 39 cases of DS samples (in a cohort of 449); however one normal sample was misclassified as DS (Table 2) [1]. The method described by Chiu *et al.* diagnosed a DS fetus when the Z-score for the proportion of chromosome 21 DNA molecules was >3, which indicates a 99% chance of statistical significance (Table 2) [8]. This method simply normalizes the number of sequence tags on the chromosome of interest by the number of tags in the sequencing run. However, it has been identified that using MPS, intra-run and inter-run variability can alter the chromosomal distribution of sequence reads for each sample. Some of the variability can come from sample handling, such as the DNA extraction procedure or the sequencing itself can lead to small shifts in the distribution of tags [88]. To minimize the intra- and inter-run sequencing variation, a study by Sehnert *et al.* developed an optimized algorithm by using normalized chromosome values (NCVs) from the sequence data [72]. When chromosome ratios are normally distributed, the NCV is equivalent to a statistical Z-score for the ratios. Threshold values for trisomy were established for all chromosomes of interest (13, 18 and 21). NCV values >4.0 were required for classification of affected aneuploidy state, and NCV values <2.5 were used to classify unaffected cases. NCV values between 2.5 and 4 were classified as “no call”. Using these parameters, this study demonstrated 100% correct classification of samples with DS and Trisomy 18. However, one sample for chromosome 13 was classified as a “no call” [72]. Some speculation exists that the poor detection rate using NGS for trisomy 13 may be due in part to the lesser level of fragmentation of this larger chromosome [89]. 

**Table 2:** Clinical trials of NIPT by massively parallel sequencing (MPS) for fetal DS (adapted from [41]).

Study	Method	DR (%)	FPR (%)
Chiu *et al*. [90]	Shotgun (2-plex protocol)	100	2.1
Chiu *et al*. [90]	Shotgun (8-plex protocol)	79.1	1.2
Ehrich *et al*. [1]	Shotgun	100	0.2
Bianchi *et al*. [91]	Shotgun	100	0
Jensen *et al*. [92]	Shotgun	100	0.9
Sparks *et al*. [93]	Targeted	100	0.8
Ashoor *et al*. [88]	Targeted	100	0
Norton *et al*. [94]	Targeted	100	0.1
Liang *et al*. [95]	Targeted	100	0

MPS technologies have successfully enabled the NIPT of fetal chromosomal aneuploidies. The identification of DS was primarily identified, and currently many recent clinical studies have indicated detection rates >99% [1,90]. The incorporation of MPS for the detection of trisomy 18 and trisomy 13 was proved to be more difficult than detecting DS due to the relatively lower GC content expressed by these two chromosomes in comparison to chromosome 21. However, when the coefficient of variance (CVs) was adjusted with GC content, it was noted that trisomy 18 and trisomy 13 can be detected accurately [87]. Chromosome 21 only represents less than 1.5% of the genome (in disomy cases) and as MPSS is not selective, millions of DNA fragments must be sequenced in order to detect statistically significant differences between trisomic and normal fetuses [96]. Therefore targeted methods have been developed, which count only specific sequences in contrast to shotgun sequencing, which counts all free DNA. In a recent statement from the Aneuploidy Screening Committee on behalf of the ISPD, it was noted that only cffDNA analysis based on MPS with either “shotgun” or “targeted” counting have been sufficiently validated to be considered analytically sound [41]. Targeted sequencing can allow for more samples to be multiplexed at once, proving a cheaper alternative to whole genome sequencing (WGS). However, the limitation of this method is that only the region of interest can be studied.

Aria Diagnostics (San Jose, CA, USA) have developed a multiplex MPS assay, termed ‘‘Digital Analysis of Selected Regions’’ (DANSR) which sequences regions from target chromosomes. In a study by Sparks *et al.* DANSR was used to develop an algorithm, the Fetal-fraction Optimized Risk of Trisomy Evaluation (FORTE), which combines both the age-related risks and the proportion of cffDNA in the samples to provide an individual risk score for trisomy. The low proportion of cffDNA within the maternal circulation can make quantification of fetal chromosome imbalances difficult and potentially inaccurate, however, the FORTE algorithm factors in the fetal fraction when calculation the risk of aneuploidy. When there is a high proportion of cffDNA the difference between trisomic *versus* disomic chromosomes is greater, making it easier to detect trisomy [93]. This approach was also reported by Ashoor *et al.* which included a cohort of 400 samples from pregnancies with known karyotypes, 300 euploid (normal), 50 trisomy 18 (Edwards syndrome) and 50 trisomy 21 (DS). Both these reports which used the DANSR/FORTE assay identified high degrees of accuracy (Table 2) [88]. However, in these trials the test was only offered to high-risk pregnancies, but the future aim is to deliver this assay to all pregnancies as a highly accurate screening test for aneuploidies [97]. Chui *et al.* identified that if referrals for amniocentesis or CVS were based on sequencing test results; approximately 98% of the invasive diagnostic procedures could be avoided [90]. In 2012 Aria Diagnostics announced the launch of a U.S. clinical study involving 25,000 pregnancies to compare FORTE with the current combined screening test for DS [98]. 

Table 3 illustrates some of the NGS platforms that are currently available. The HiSeq2000 has a significantly higher number of single end reads per run, which makes this platform very suitable for multiplexing samples and thus high throughput runs. However with the development of targeted counting smaller bench-top platforms such as the MiSeq and Ion Torrent could be used for more rapid testing due to reduced sample-prep time and faster run times, however these platforms will exert lower throughput due to lower number of single end reads per run. Even though the initial costs are cheaper for the bench-top platforms (MiSeq and Ion Torrent), because of the increased number of base reads per run with high throughput platforms (HiSeq2000), the cost per Mb is actually cheaper for the HiSeq2000 ($0.07) than the Illumina MiSeq ($0.5) and Ion Torrent ($0.64) [99,100]. 

**Table 3:** NGS Platforms suitable for NIPT (adapted from [96,101]).

	PCR-based sequencing	Single end reads per run	Run Time
**HiSeq™2000** (Illumina, Inc.)	Sequencing-by-synthesis	3 billion	5–14 days
**HiSeq™2500** (rapid run) (Illumina, Inc.)	Sequencing-by-synthesis	~300 million (10 Gb)	7 h
**SOLiD4™** (Life Technologies™/Applied Biosystems ™)	Sequencing-by-ligation	~0.7 billion	5–10 days
**HeliScope® Single Molecule Sequencer** (Helicos™Biosciences)	Single-molecule-sequencing-by-synthesis	~840 million (28 Gb)	8 days
Benchtop: **MiSeq™** (Illumina, Inc.)	Sequence-by-synthesis	~12 million (3.4 Gb)	16.5 h
Benchtop: **Ion Torrent™** (Life Technologies™)	Semiconductor sequencing technology	~5 million (1 Gb)	4.4 h

The International Society for Prenatal Diagnosis (ISPD) has reported that before routine MPS population screening can be introduced additional trials are needed. These trials need to confirm that there is efficacy in low-risk populations, that it is cost-effective and suitable for diverse subpopulations (such as twin or IVF pregnancies) [102]. Commercial MPS-based testing for prenatal detection of DS has been introduced into some areas of the United States, China and more recently the European Union (EU). Currently there are three commercial providers of NIPT within the USA who have received Clinical Laboratory Improvement Amendments (CLIA) certification (Table 4); however more recently an additional competitor, Natera, has entered the market (Table 4) [103,104]. The Harmony test (provided by Aria Diagnostics) is currently the cheapest ($795); however this test uses selective sequencing in comparison to the Verifi test and the MaterniT21 Plus test, which use shotgun sequencing for aneuploidy detection. According to a study published earlier this year, with reference to WGS, the sequencing alone can already be done for less than $1,000, however soon it is likely the entire process will drop below the $1,000 mark [105]. The ISPD has outlined that this NIPT should be offered to high-risk pregnancies only and not offered as an initial test as screening via MPS for all pregnancies as it is not currently cost effective [102]. It is vital that all women undergoing MPS-based testing are offered prenatal counseling, so that the benefits and limitations of the test can be explained. 

**Table 4:** Commercial tests available for the NIPT of trisomies (adapted from [104]).

Company	Test	Released	Trisomies Tested	Genetic Testing Method	Accuracy	Sensitivity	Cost
Sequenom	MaterniT21 Plus	February 2012	13, 18, 21, sex chromo-somes	MPSS	>99%	92–99%	$2,762
Verinata	Verifi Prenatal Test	March 2012	13, 18, 21, sex chromo-somes	MPSS	100%	87–99%	$1,500
Aria Diagnostics	Harmony Prenatal Test	May 2012	13, 18, 21	Chromosome-selective sequencing	>99%	80–99%	$795
Natera	Panorama	March 2013	13, 18, 21	Single nucleotide polymorphism	100%	92–99%	$1,495

## 5. Conclusions

This review demonstrates how screening for the detection of DS has improved since the early 1980s when maternal age was the only “tool” available. It also provides an insight into how new physical and biochemical markers may play a role in future routine screening to allow for increased test sensitivity with fewer false screen positives. Although this is still a key area of research, the main focus is to provide a definitive diagnosis through non-invasive techniques, such as digital PCR and NGS. A recent trial conducted within the UK to assess the performance of NIPT for fetal trisomy in a routinely screened first-trimester population identified a DR of >99% and a false positive rate of 0.1% for trisomy 21 and trisomy 18, which is a significant improvement on current screening DRs and FPRs (85–90% and 5%, respectively) [50,106]. Although the sensitivity of NGS currently provides DRs similar to that provided by CVS, before even considering the replacement of IPD with NIPD, further large scale validation studies of low-risk populations are required to confirm that NGS test sensitivity is consistent with current invasive testing (97.8% and 99.4% for CVS and amniocentesis, respectively) [107]. It is also important that the economic aspects, counseling requirements and turnaround times are also considered [97]. 

The cost of NIPD is likely to vary by country due to variations in the accuracy of the NIPD test, the cost of the NIPD test and the numbers undergoing NIPD [97]. However for fetal aneuploidy testing, whole genome MPS is still quite expensive, therefore to lower costs targeted approaches are being developed [96]. Using MPS in high-risk pregnancies following initial screening increases the OAPR, causing fewer women with unaffected fetuses to miscarry. Furthermore, providing NGS to all pregnancies would not only increase the OAPR but also reduce the number of unidentified trisomy fetuses, however this would be associated with a dramatic increase in cost due to a substantial rise in the numbers undergoing NIPD. 

Developments in proteomics to detect multiple novel biomarkers could provide a cheaper screening alternative to NGS but will most likely display a reduction in sensitivity. However, new biomarkers can only be used for screening purposes, whereas MPS directly identifies fetal DNA providing a NIPD approach that could potentially replace current IPD techniques. With the continuous decline in MPS costs, NIPD of fetal aneuploidy is an exciting area of research that could become a clinical reality for all pregnancies in the near future. 
